# Cardiopulmonary exercise testing early after stroke using feedback-controlled robotics-assisted treadmill exercise: test-retest reliability and repeatability

**DOI:** 10.1186/1743-0003-11-145

**Published:** 2014-10-11

**Authors:** Oliver Stoller, Eling D de Bruin, Matthias Schindelholz, Corina Schuster-Amft, Rob A de Bie, Kenneth J Hunt

**Affiliations:** Department of Engineering and Information Technology, Institute for Rehabilitation and Performance Technology, Bern University of Applied Sciences, Burgdorf, Switzerland; Department of Epidemiology, Maastricht University and Caphri Research School, Maastricht, Netherlands; Research Department, Reha Rheinfelden,, Rheinfelden, Switzerland; Department of Health Sciences and Technology, Institute of Human Movement Sciences and Sport, ETH Zurich, Zurich, Switzerland; Centre for Evidence Based Physiotherapy, Maastricht University, Maastricht, Netherlands

**Keywords:** Stroke rehabilitation, Subacute, Severe motor impairment, Robotics-assisted gait training, Aerobic capacity, Treadmill exercise

## Abstract

**Background:**

Exercise capacity is seriously reduced after stroke. While cardiopulmonary assessment and intervention strategies have been validated for the mildly and moderately impaired populations post-stroke, there is a lack of effective concepts for stroke survivors suffering from severe motor limitations. This study investigated the test-retest reliability and repeatability of cardiopulmonary exercise testing (CPET) using feedback-controlled robotics-assisted treadmill exercise (FC-RATE) in severely motor impaired individuals early after stroke.

**Methods:**

20 subjects (age 44–84 years, <6 month post-stroke) with severe motor limitations (Functional Ambulatory Classification 0–2) were selected for consecutive constant load testing (CLT) and incremental exercise testing (IET) within a powered exoskeleton, synchronised with a treadmill and a body weight support system. A manual human-in-the-loop feedback system was used to guide individual work rate levels. Outcome variables focussed on standard cardiopulmonary performance parameters. Relative and absolute test-retest reliability were assessed by intraclass correlation coefficients (ICC), standard error of the measurement (SEM), and minimal detectable change (MDC). Mean difference, limits of agreement, and coefficient of variation (CoV) were estimated to assess repeatability.

**Results:**

Peak performance parameters during IET yielded good to excellent relative reliability: absolute peak oxygen uptake (ICC =0.82), relative peak oxygen uptake (ICC =0.72), peak work rate (ICC =0.91), peak heart rate (ICC =0.80), absolute gas exchange threshold (ICC =0.91), relative gas exchange threshold (ICC =0.88), oxygen cost of work (ICC =0.87), oxygen pulse at peak oxygen uptake (ICC =0.92), ventilation rate versus carbon dioxide output slope (ICC =0.78). For these variables, SEM was 4-13%, MDC 12-36%, and CoV 0.10-0.36. CLT revealed high mean differences and insufficient test-retest reliability for all variables studied.

**Conclusions:**

This study presents first evidence on reliability and repeatability for CPET in severely motor impaired individuals early after stroke using a feedback-controlled robotics-assisted treadmill. The results demonstrate good to excellent test-retest reliability and appropriate repeatability for the most important peak cardiopulmonary performance parameters. These findings have important implications for the design and implementation of cardiovascular exercise interventions in severely impaired populations. Future research needs to develop advanced control strategies to enable the true limit of functional exercise capacity to be reached and to further assess test-retest reliability and repeatability in larger samples.

**Electronic supplementary material:**

The online version of this article (doi:10.1186/1743-0003-11-145) contains supplementary material, which is available to authorized users.

## Background

Exercise capacity and activity status have become well-established predictors of cardiovascular and overall mortality, both of which are seriously reduced after stroke [1, 2]. It has been shown that peak oxygen uptake (VO_2_peak) is approximately 50% lower compared to normative values of healthy adults 30 days post-stroke [3, 4]. Despite extensive inpatient rehabilitation procedures and spontaneous recovery of cardiovascular fitness, the exercise capacity of stroke survivors entering the chronic phase remains below recommended levels [5]. The rapid deterioration of fitness not only predisposes to secondary medical complications, but also restricts the degree to which individuals can participate in rehabilitation routines and limits the ability of the individual to perform functional activities independently [6]. Therefore, research into cardiovascular exercise training in the early stages after stroke has been highlighted as a priority [7, 8]. Effective assessment and intervention strategies are needed to assess, monitor, and improve cardiovascular fitness early after stroke.

Current research has investigated several modalities for cardiopulmonary exercise testing (CPET) in subacute stroke (6 days-6 months post-stroke) [3, 4, 9–13] and in chronic stroke (>6 months post-stroke) [14–19] using treadmill exercise [14–16], body weight supported treadmill exercise [3], leg cycle ergometry [4, 9–11, 15, 17, 18], and combined upper- and lower-limb ergometry [12, 19]. The most common concepts, i.e. treadmill exercise and leg cycle ergometry, are primarily designed for individuals with mild to moderate motor impairment, because limited motor control (non-ambulatory status, limited trunk control), poor postural control, and poor coordination of the affected limbs may restrict severely impaired individuals from performing on these devices. As a result, most studies focussing on exercise capacity after stroke have excluded individuals not able to walk independently and those presenting with low levels of motor function.

A potential option to overcome severe motor restrictions is the introduction of combined upper- and lower-limb ergometry [12, 19, 20]. Current study results demonstrated feasibility and validity, and emphasised the fact that an all-extremity exercise protocol might decrease early onset of lower limb fatigue which leads to better estimates of exercise capacity due to the incorporation of more muscle mass. However, this approach does not embody the concept of repetitive task-specific exercise during the early stages of stroke recovery and might be not appropriate for implementation into early rehabilitation phases [21, 22]. Considering the relatively short intervention window during subacute stroke rehabilitation and the current recommendations for cardiovascular exercise training after stroke [8], novel approaches should incorporate task-specific activities such as walking or stair climbing. The combination of motor function training and cardiovascular exercise might have the potential to positively influence overall therapy outcomes and to prevent or mitigate the loss of exercise capacity in the early stages after stroke onset [23].

A promising approach to overcome motor limitations while facilitating task-specific activity and cardiovascular stress is body weight supported treadmill training. Initial research has shown that gait symmetry improved with increasing body weight support (BWS) [24]. However, during walking with BWS of more than 15%, vertical ground reaction forces and functional activity of antigravity muscles decreased, which led to substantially lower oxygen uptake levels during body weight supported treadmill training compared to conventional treadmill exercise [25, 26]. Because severely impaired stroke survivors need considerable physical support during walking with low body weight support, the application of robotics-assisted treadmill exercise (RATE) might be of relevance in this context. A powered exoskeleton for the lower extremities, synchronised with a treadmill and BWS, provides active support during the gait trajectory that enables progressive body weight loading for individuals with severe motor restrictions.

Recent research on exercise intensity during RATE has shown substantial increases in cardiopulmonary performance parameters after stroke [27, 28], and spinal cord injury [29], including complete tetraplegia [30]. However, oxygen uptake levels were below that of overground walking, recommended cardiovascular training intensities could not be achieved [31], and conventional control strategies such as the modulation of walking speed, BWS, and guidance force had only a minor influence on exercise intensity [27, 28, 31]. There is a need for voluntary effort during walking within an exoskeleton to provoke substantial cardiovascular stress comparable to conventional treadmill exercise [32]. Therefore, novel protocols have been developed to control and direct active participation during RATE with the specific aim of provoking cardiorespiratory responses [33–38]. This incorporates biofeedback mechanisms allowing the control of exercise intensity through the guidance of the individual’s voluntary effort. The approach presented here provides control of exercise intensity during RATE by biofeedback and voluntary adaptation of the hip and knee forces by the subject. A first clinical study in non-ambulatory stroke survivors in the subacute phase revealed that feedback-controlled RATE (FC-RATE) can be used to implement CPET [39]. Results yielded acceptable cardiopulmonary performance parameters following standardized CPET protocols. Thus, this approach might have the potential to assess exercise capacity and guide cardiovascular exercise in stroke survivors with severe motor limitations. This needs to be formally investigated for clinical feasibility, test-retest reliability and repeatability.

The aims of this study were: (1) to assess the clinical feasibility of FC-RATE for CPET in severely motor impaired individuals early after stroke, (2) to examine the ability of the concept to meet standard cardiopulmonary criteria for maximal exercise capacity, and (3) to assess the test-retest reliability and the repeatability of the approach.

## Methods

### Participants

20 first-ever stroke inpatients were recruited at a neurological rehabilitation clinic in the north-western part of Switzerland (Reha Rheinfelden) and screened according to the selection criteria. Subjects were then presented to the responsible ward physician and a cardiologist to confirm eligibility. Inclusion criteria were: (1) clinical diagnosis of initial stroke (ischemic or haemorrhagic), (2) <20 weeks after stroke onset, (3) age >18 years, (4) Functional Ambulation Classification (FAC) of <3, (5) ability to understand the procedures and provide informed consent. Subjects were excluded if they had (1) cardiac contraindications for exercise testing according to the American College of Sports Medicine (ACSM) [40], (2) contraindications for RATE according to guidelines from the manufacturer (Hocoma AG, Volketswil, Switzerland), (3) concurrent neurological disease (e.g. Multiple Sclerosis, Parkinson’s Disease, etc.), (4) concurrent pulmonary disease (e.g. COPD, etc.), (5) history of dementia.

Recorded characteristics included gender, age, body mass index, diagnosis, affected body side, time post-stroke, medications, comorbidities, FAC [41] and functional independence using the Extended Barthel Index [42]. All subjects were informed about risks and benefits, and gave signed informed consent. The Ethics Review Committee of the Swiss canton of Aargau approved the study (Reference No: 2012/051).

### Technical implementation

**Figure 1:**
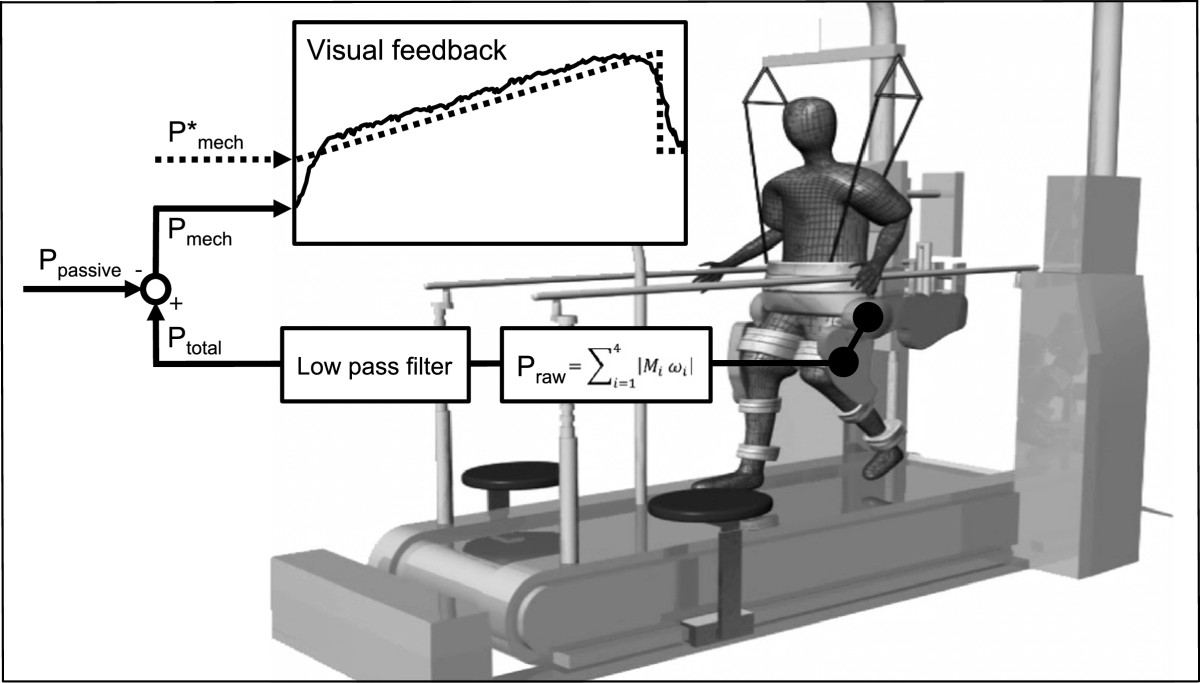
**Feedback-controlled robotics-assisted treadmill exercise.** Hip and knee joint forces and angles are measured in real time to allow calculation of the mechanical work rate (Pmech, solid line) and projection onto a screen in front of the subject. Individual target work rate profiles (P*mech, dashed line) are used to guide exercise intensity during robotics-assisted walking. The passive mechanical work rate (Ppassive) is evaluated before every session and subtracted from Pmech. Legend: Praw = raw mechanical work rate, Μ_i_ = moments of force, ω_i_ = angular velocity, Ptotal = total mechanical work rate.

The Lokomat system (Hocoma AG, Volketswil, Switzerland) was used to implement FC-RATE. The powered exoskeleton provides control of both legs using DC motors, synchronised with an integrated treadmill (h/p/cosmos sports & medical GmbH, Traunstein, Germany) and a motor-driven BWS system with real time feedback control for precise body weight unloading (Lokolift, Hocoma AG). The total mechanical work rate exerted on the exoskeleton by the subject was computed from the force, moment arm and velocity data at the four active joints (hips and knees). The active mechanical work rate (Pmech), applied by the subject’s effort was estimated by subtracting the passive mechanical work rate (work rate necessary to move the subject passively within the exoskeleton) from the total mechanical work rate. A manual human-in-the-loop feedback system was implemented to control the subject’s active work rate. Pmech was projected onto a screen at the front of the treadmill together with a target mechanical work rate (P*mech). The subject was instructed to vary the forces applied on the exoskeleton by volitional muscle activity and to keep the measured and visualized active work rate as close as possible to the target (Figure 1).

### Experimental protocol

At study entry, all included subjects completed a familiarisation session with the FC-RATE concept, which started by qualified and experienced physiotherapists adjusting the Lokomat system to provide a physiological gait pattern and to ensure that the subjects could walk comfortably. Then, an initial test of decreasing BWS continuously by 5% per minute was implemented to define the minimal possible BWS level. There was strict adherence to physiological gait pattern criteria through visual observation: (1) heel strike (physiological knee extension), (2) no foot dragging during the swing phase, and (3) active weight-bearing during the stance phase (physiological knee extension) [43]. After the first adjustments, subjects were asked to perform a short constant load exercise test for 5 min (P*mech =20 W) to explain the approach and practice with the feedback-control structure. Finally, the safety procedures for potential adverse events were explained in detail.

After a break of at least 24 h, subjects then completed repeated constant load testing (CLT) and incremental exercise testing (IET) on separate days, with 48–72 h between the trials. All sessions were controlled for time of day. Subjects were instructed to avoid additional strenuous activity during participation in the study and not to consume food, alcohol, nicotine or caffeine at least 3 h prior to testing.

Subjects were asked at the beginning of the first CLT and IET to increase their maximal voluntary effort during RATE within 30 s to define the maximal work rate (Pmax) for the subsequent tests. Walking cadence was fixed at 60 steps/min and individual BWS was consistent for all sessions. An experienced examiner performed all tests. There was close adherence to established models for exercise testing according to the ACSM guidelines [40].

**Figure 2:**
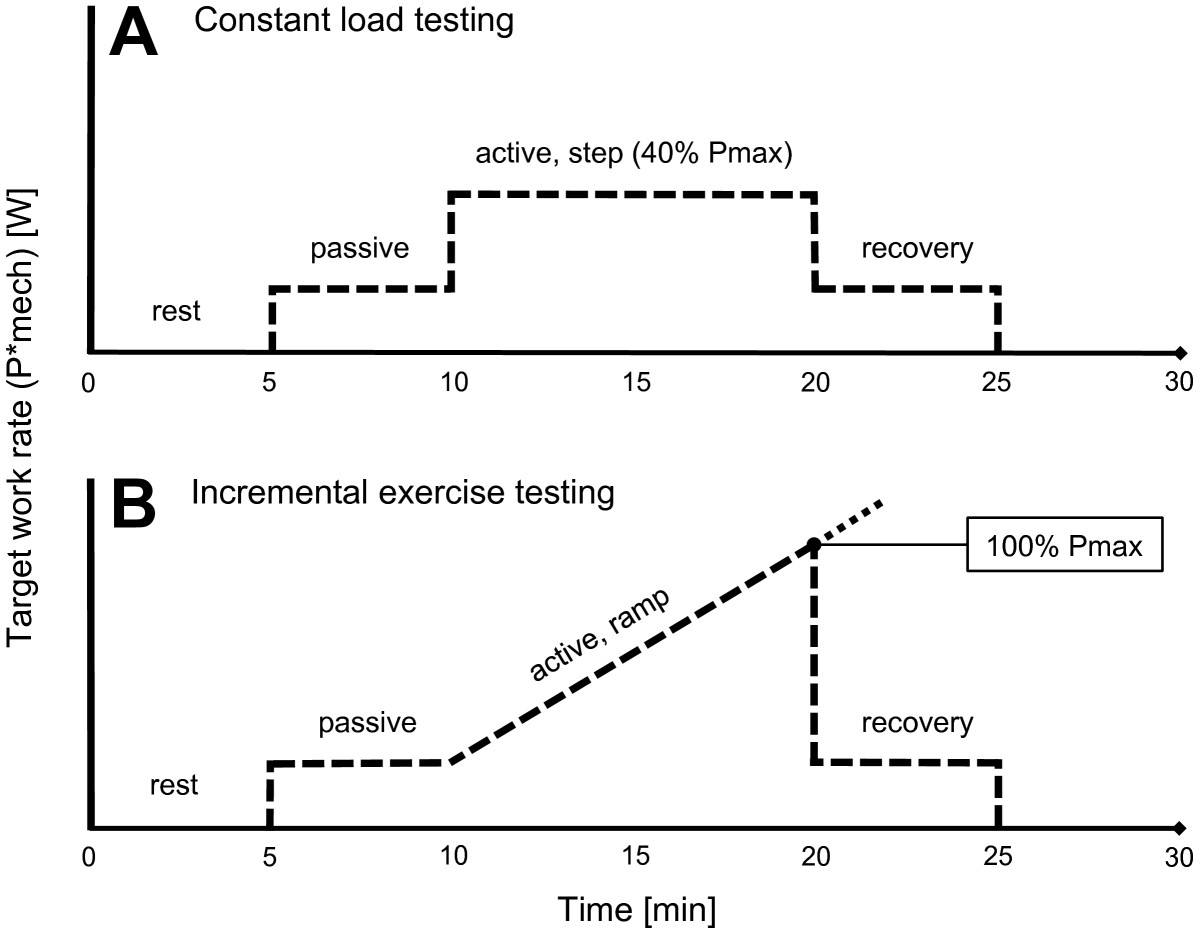
**Exercise testing protocols.** Schematic representation of constant load testing, CLT, **(A)** and incremental exercise testing, IET, **(B)** using feedback-controlled robotics-assisted treadmill exercise. The dashed line represents the target work rate (P*mech). The slope during incremental exercise testing was estimated such that the predefined work rate maximum (Pmax) was reached at 10 min during the active phase. When individual termination criteria were met the incremental phase was ended and P*mech set back to the passive level (recovery).

CLT was based on constant-intensity exercise (40% Pmax) separated into 4 phases: (1) rest - subjects stood on the treadmill for 5 min with 0% BWS, (2) passive phase - subjects walked passively with their individual BWS for 5 min, (3) active phase - subjects actively contributed to the walking by pushing forward within the exoskeleton during the swing phase of each leg to reach the target work rate for 10 min, (4) recovery - subjects walked passively with their individual BWS for 5 min (Figure 2A).

IET was based on progressive ramp exercise and separated into 4 phases: (1) rest - subjects stood on the treadmill for 5 min with 0% BWS, (2) passive phase - subjects walked passively with their individual BWS for 5 min, (3) active phase - subjects actively contributed to the walking by pushing forward within the exoskeleton during the swing phase of each leg to reach the target work rate, (4) recovery - subjects walked passively with their individual BWS for 5 min. The progressive ramp (active phase) was defined as a continuous slope aiming to the reach predefined Pmax in 10 min (Figure 2B).

Both test protocols followed strict termination criteria for CPET including: (1) abnormal blood pressure responses, i.e. hypertensive (systolic >210 mmHg/diastolic >115 mmHg) when exercising at high work rate, or hypotensive responses (decrease of >10 mmHg) despite an increase in work rate, (2) individual work rate below target work rate for 60 s, (3) peak heart rate within 10 beats per minute of the age-predicted heart rate maximum [44], where the formula was adjusted down to 70% of heart rate maximum for subjects on beta-blocker medications [45], (d) pain or discomfort. Subjects rated their perceived exertion using the Borg rating of perceived exertion scale (RPE) (6 = no exertion at all, 20 = maximal exertion) [46].

Several risk management strategies were implemented to ensure subjects’ safety: (1) clearly defined eligibility criteria to include medically stable subjects only, (2) screening by cardiologists to exclude subjects with potential risk factors (i.e. abnormalities in resting ECG, history of any cardiac/cardiovascular disease, uncontrolled metabolic disease), (3) continuous blood pressure and heart rate monitoring during exercise testing, (4) presence of resuscitation-trained assistants, (5) opportunity to call the emergency medical resuscitation team in the clinic, and (6) presence of personnel trained to release the subject within 60 s from the exoskeleton. Detailed information on FC-RATE-based CPET can be found elsewhere [39, 47].

### Outcomes

Measured cardiopulmonary performance parameters were: oxygen uptake (VO_2_), carbon dioxide output (VCO_2_), ventilation rate (V_E_), respiratory rate (R_f_), and heart rate (HR). These were recorded by a breath-by-breath cardiorespiratory monitoring system (MetaMax 3B, Cortex Biophysik, Leipzig, Germany), including a heart rate belt (T31, Polar Electro, Kempele, Finland) and a receiver board (HRMI, Sparkfun, Boulder, USA). Pmech was calculated using the exoskeleton geometry and interaction forces, and angular signals, which were available in real time from a custom interface unit.

For CLT, outcome variables were speed of oxygen uptake kinetics (time constant τ), oxygen cost of passive walking (Δ rest vs. passive walking), oxygen cost of active walking (Δ passive walking vs. active walking), and accuracy of work rate tracking (RMSE_P_). IET focused on peak performance parameters for oxygen uptake (VO_2_peak), time to VO_2_peak (tVO_2_peak), work rate (Ppeak), ventilation rate (V_E_peak), respiratory rate (R_f_peak), heart rate (HRpeak), and respiratory exchange ratio (RERpeak). In addition, gas exchange threshold (GET), oxygen cost of work (ΔVO_2_/ΔP), O_2_ pulse at VO_2_peak (O_2_pulse), V_E_ versus VCO_2_ slope (ΔV_E_/ΔVCO_2_), and RMSE_P_ were evaluated.

### Data processing

Raw breath-by-breath data were processed using a zero phase shift moving average filter over 15 breaths [48]. For CLT, the time constant for the oxygen uptake kinetics (τ) was calculated using a non-linear least-squares algorithm to fit the data as described in the following mono-exponential equation: VO_2_(t) = VO_2_(b) + ΔVO_2_(1 ‒ e^‒ (t ‒ Td)/τ^), t > 0, with VO_2_(b) = oxygen uptake at baseline, ΔVO_2_ = step increase in oxygen uptake, Td = time delay of 20 s corresponding to the cardio-dynamic phase of the response, and τ = time constant [49]. Steady-state was defined by excluding the first 2 minutes and last minute of each phase, i.e. steady-state calculations were done using data from the 3^rd^ – 4^th^ minute of a given phase. Cost of passive walking was defined as the difference between rest and passive steady-state values, whereas cost of active walking was estimated from the difference between passive and active steady-states. For IET, peak cardiopulmonary response variables were defined as the maximal values in the final 30 s during the incremental phase. Criteria for maximal aerobic capacity were (1) plateau in oxygen uptake, (2) respiratory exchange ratio (RER) ≥1.15, and (3) peak heart rate within 10 beats per minute of the age-predicted heart rate maximum (adjusted for subjects on beta-blocker medications) [40]. The identification of a plateau or reduction in VO_2_ was performed by plotting the slope and 95% confidence interval (CI) of the VO_2_-Pmech slope by least-squares linear regression analysis, where the presence of data points that fell below and outside the extrapolated 95% CI were taken as evidence of plateauing or levelling-off behaviour [50]. The GET was estimated using the v-slope method, where the anaerobic threshold is identified as the deflection point of the VO_2_-VCO_2_ relationship [51]. The accuracy of work rate tracking (RMSE_P_) was expressed by the root mean square error between Pmech and P*mech. Data processing was performed using MATLAB (Version R2010a, MathWorks, Natick MA, USA) and LabVIEW (Version 2009, National Instruments, Austin TX, USA).

### Statistical analysis

Descriptive statistics were calculated for all outcome variables. Due to the small sample size, Wilcoxon-tests were applied to exclude significant practice effects. Test-retest reliability was quantified using intraclass correlation coefficients (ICC_3,1_) with 95% CI. The ICC provides an estimate of the relative reliability of measurement when the population under study is heterogeneous [52]. ICC results of 0.60-0.74 were considered as “good”, and ICC results >0.74 as “excellent” [53]. Absolute reliability was determined by estimating the standard error of measurement (SEM = standard deviation of the difference $\left(\mathrm{SDdiff}\right)\sqrt{1‒ICC}$) and the minimal detectable change $\left(MDC\;=\;1.96\times \sqrt{2}\times SEM\right)$, presented in absolute values and percentages [54, 55]. Repeatability was estimated by mean difference (MD), limits of agreement (LoA) (MD ±1.96 x SDdiff), and coefficients of variation (CoV) (SDdiff/mean). Two-sided p-values p ≤0.05 were considered significant. Statistical analyses were performed using SPSS (Version 20.0, IBM, Armonk NY, USA) and MATLAB (Version R2010a, MathWorks, Natick MA, USA).

## Results

### General observations

**Figure 3:**
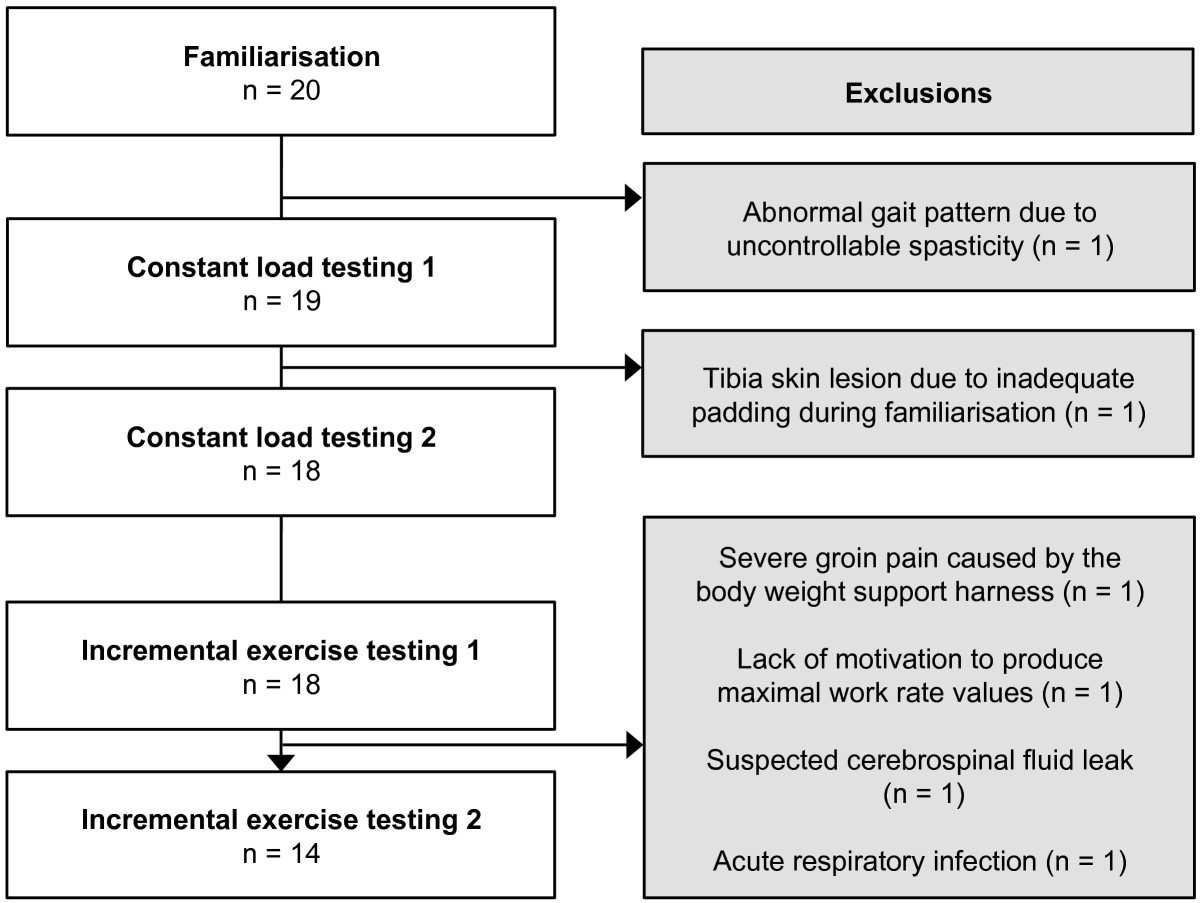
**Study flow chart.**

Of the 20 subjects enrolled in the study, 1 subject showed an abnormal gait pattern due to uncontrollable spasticity during familiarisation and 1 subject developed a tibia skin lesion due to inadequate padding of the exoskeleton, which led to withdrawal from the study (Figure 3). Further, 4 withdrawals after the first IET occurred due to groin pain, lack of motivation, suspected cerebrospinal fluid leak, and acute respiratory infection. Thus, 18 subjects (90%) performed the two CLTs and, of these, 14 (70%) also performed the two IETs. All subjects presented with severe motor impairments and were non-ambulatory (FAC range 0–2). BWS ranged between 46-77% and walking speed was set at 60 steps/minute (0.47-0.67 m/s). The subject characteristics are summarized in Table 1.

**Table 1:** **Subject characteristics**

	Constant load testing	Incremental exercise testing
	(n =18)	(n =14)
Men/women	11/7	9/5
Type of stroke: ischemic/haemorrhagic	12/6	11/3
Hemiparetic side: right/left	9/9	8/6
Time post-stroke [d]	49±31(14–139)	43±25(14–92)
Age [y]	61±11(44–84)	61±12(44–84)
BMI [kg/m^2^]	27±5(19–38)	28±6(19–38)
FAC (0–5)	1.1±0.8(0–2)	0.9±0.8(0–2)
EBI (0–64)	43±9(27–56)	42±9(27–55)
Medications: beta-blockers/ACE inhibitors/both	6/11/4	4/11/4
Comorbidities: Hypertension/Dyslipidemia/Adipositas/Diabetes mellitus	9/6/3/3	8/6/3/3
BWS [%]	59±9(46–77)	60±9(46–77)
Walking speed [m/s]	0.57±0.05(0.47-0.67)	0.56±0.05(0.47-0.64)
RPE (6–20)	13±2(6–17)	15±2(11–18)
Pmax [W]	38.4±23.0(8.5-77.2)	57.1±33.1(11.3-127.7)

Values are given in numbers (n) or mean ± standard deviation (range).

*Abbreviations: BMI* Body mass index, *FAC* Functional Ambulation Classification, *EBI* Extended Barthel Index, *ACE inhibitors* Angiotensin-converting enzyme inhibitors, *BWS* Body weight support, *RPE* Rate of perceived exertion, *Pmax* Maximal work rate.

All subjects successfully completed the predefined CPET protocol (rest, passive, active, recovery). During CLT, 3 subjects stopped the active walking phase after 5 minutes due to generalized fatigue and continued with the recovery phase. All other CLT conditions were performed according to the plan. 13 subjects completed both IETs without symptomatic responses requiring termination per safety criteria; the reason for test termination was in every case the inability to reach P*mech due to generalized and/or leg fatigue. The examiner stopped 2 consecutive IET sessions in 1 subject due to high blood pressure responses according to the safety criteria (2 adverse events); however, no serious adverse events occurred during testing. Mean IET duration was 23.2 ± 2.6 min (active phase: 8.2 ± 2.6 min). RPE at peak performance was 14.8 ± 1.9. RMSE_P_ values were <10 W.

### Exercise capacity

For CLT, time constants of oxygen uptake kinetics (τ) could not be evaluated due to continuous disturbances of VO_2_ in the transition phases (rest/passive walking, passive walking/active walking). Cost of passive walking (Δ rest vs. passive walking) was (mean ± standard deviation): VO_2_ =184.2 ± 124.0 mL/min, HR =1.4 ± 6.4 beats/min, and cost of active walking (Δ passive walking vs. active walking) was: VO_2_ =45.7 ± 56.6 mL/min, HR =2.3 ± 3.1 beats/min. RMSE_P_ during CLT was 5.5 ± 5.5 W. For IET, peak performance parameters were: absolute VO_2_peak =1280.9 ± 564.8 mL/min, relative VO_2_peak =15.5 ± 4.9 mL/min/kg (51.6 ± 20.5% of predicted VO_2_max [56]), tVO_2_peak =8.2 ± 2.6 min, Ppeak =53.9 ± 33.8 W, V_E_peak =41.3 ± 18.6 L/min, R_f_peak =36.1 ± 8.3 1/min, HRpeak =126.0 ± 19.5 beats/min (84.3 ± 12.2% of age-predicted heart rate maximum [44]), and RERpeak =0.92 ± 0.09. Absolute GET was at a VO_2_ of 878.9 ± 316.6 mL/min and relative GET at 11.0 ± 3.1 mL/min/kg, which was GET% =72.6 ± 12.2% of VO_2_peak. ΔVO_2_/ΔP was 20.1 ± 14.4 mL/W, O_2_pulse was 10.2 ± 4.1 mL/beat, and ΔV_E_/ΔVCO_2_ was 36.3 ± 7.3 L. RMSE_P_ during IET was 8.7 ± 9.0 W.

With respect to the 3 criteria for maximal aerobic capacity, 2 subjects (14%) showed a plateau in VO_2_ at the end of IET, 1 subject (7%) achieved an RER value ≥1.15, and 5 subjects (36%) reached peak heart rate within 10 beats per minute of the age-predicted heart rate maximum, where 2 of these subjects had an adjusted heart rate due to beta-blockers. Thus, 57% of the subjects achieved at least 1 of the 3 criteria for maximal exercise capacity.

### Test-retest reliability and repeatability

Table 2 shows mean values, test-retest reliability and repeatability results of the repeated CLT and IET trials. No practice effects could be detected; trials were not significantly different. Outcome variables for CLT yielded high MD between tests and insufficient test-retest reliability and repeatability throughout. For IET, good to excellent relative reliability was found for absolute VO_2_peak (ICC =0.82), relative VO_2_peak (ICC =0.72), Ppeak (ICC =0.91), HRpeak (ICC =0.80), absolute GET (ICC =0.91), relative GET (ICC =0.88), ΔVO_2_/ΔP (ICC =0.87), O_2_pulse (ICC =0.92), and ΔV_E_/ΔVCO_2_ (ICC =0.78). SEM were between 4-13% and MDC ranged from 12-36%. MD ± SDdiff of the outcome variables that were analysed for relative reliability were: absolute VO_2_peak =45.5 ± 353.7 mL/min, relative VO_2_peak =1.0 ± 3.8 mL/min/kg, Ppeak =2.4 ± 15.0 W, HRpeak =3.6 ± 12.6 beats/min, absolute GET =67.3 ± 124.2 mL/min, relative GET =0.2 ± 1.6 mL/min/kg, ΔVO_2_/ΔP =2.7 ± 7.2 mL/min/W, O_2_pulse =0.1 ± 1.7 mL/beat, ΔV_E_/ΔVCO_2_ =0.5 ± 5.1 L. CoV for peak cardiopulmonary performance parameters ranged from 0.10-0.44. Bland–Altman plots for the major outcome variables visualize the differences between tests (Additional files 1, 2).

**Table 2:** **Test-retest reliability and repeatability of feedback-controlled robotics-assisted treadmill exercise based cardiopulmonary exercise testing**

	Trial 1		Trial 2											
	mean±SD	(range)	mean±SD	(range)	*p*-value	MD	(LoA)	CoV	ICC	(95% CI)	SEM	SEM%	MDC	MDC%
**Constant load testing (n =18)**														
VO_2_ cost of passive walking [mL/min]	200.4±112.0	(41.4-426.4)	167.9±136.2	(−3.7-506.5)	0.23	32.5	(−182.4, 247.5)	0.58	0.62	(0.25-0.84)	66.3	36	183.7	100
VO_2_ cost of active walking [mL/min/W]	43.4±53.0	(−2.5-212.8)	48.1±61.4	(2.0-214.2)	0.62	4.6	(−141.2, 150.5)	1.59	0.20	(−0.31-0.61)	65.2	143	180.8	395
Heart rate cost of passive walking [beats/min]	0.6±5.9	(−14.5-7.8)	2.3±6.9	(−13.0-15.4)	0.68	1.6	(−13.2, 16.5)	5.20	0.33	(−0.15-0.68)	6.1	426	16.9	1182
Heart rate cost of active walking [beats/min/W]	2.4±3.6	(0.0-15.5)	2.1±2.5	(0.3-9.3)	0.91	0.3	(−7.1, 7.6)	1.62	0.32	(−0.18-0.68)	3.0	134	8.4	370
Deviation of work rate (RMSE_P_) [W]	5.4±5.3	(1.4-20.9)	5.5±5.8	(0.9-21.4)	0.95	0.0	(−11.2, 11.3)	1.03	0.50	(0.05-0.78)	4.0	73	11.0	201
**Incremental exercise testing (n =14)**														
Peak VO_2_ uptake (VO_2_peak absolute) [mL/min]	1258.1±612.1	(460.3-2490.3)	1303.6±535.5	(583.2-2427.8)	0.47	45.5	(−662.0, 753.0)	0.28	0.82	(0.53-0.94)	150.5	12	417.1	33
Peak VO_2_ uptake/body mass (VO_2_peak relative) [mL/min/kg]	15.0±4.8	(7.2-23.4)	15.9±5.2	(9.1-27.9)	0.33	1.0	(−6.7, 8.6)	0.25	0.72	(0.33-0.89)	2.0	13	5.6	36
Time to VO_2_peak (tVO_2_peak) [min]	7.5±1.7	(4.9-10.8)	8.8±3.2	(4.1-16.9)	0.14	1.3	(−4.2, 6.8)	0.34	0.39	(−0.09-0.74)	2.2	26	6.0	73
Peak work rate (Ppeak) [W]	52.7±33.2	(11.3-107.4)	55.1±35.6	(7.9-101.0)	0.64	2.4	(−27.7, 32.5)	0.28	0.91	(0.74-0.97)	4.5	8	12.6	23
Peak ventilation rate (V_E_peak) [L/min]	40.3±18.6	(15.7-82.8)	42.4±19.3	(21.7-96.7)	0.73	2.1	(−34.5, 38.6)	0.44	0.55	(0.33-0.83)	12.3	30	34.1	82
Peak respiratory rate (R_f_peak) [1/min]	36.5±9.7	(22.7-54.7)	35.8±7.0	(23.9-44.3)	0.73	0.7	(−14.0, 15.4)	0.20	0.64	(0.18-0.87)	4.4	12	12.2	34
Peak heart rate (HRpeak) [beats/min]	124.2±17.6	(97–148)	127.9±21.8	(95–160)	0.35	3.6	(−21.6, 28.9)	0.10	0.80	(0.49-0.93)	5.7	5	15.8	13
Peak respiratory exchange ratio (RERpeak)	0.91±0.05	(0.84-1.00)	0.93±0.11	(0.79-1.21)	0.55	0.01	(−0.18, 0.21)	0.11	0.39	(−0.18-0.76)	0.08	8	0.21	23
Gas exchange threshold (GET absolute) [mL/min]	911.3±365.1	(323.8-1642.7)	844.0±265.0	(491.2-1255.1)	0.51	67.3	(−181.0, 315.6)	0.14	0.91	(0.74-0.97)	36.6	4	101.5	12
GET/body mass (GET relative) [mL/min/kg]	11.1±3.1	(5.1-14.7)	10.8±3.1	(5.3-16.6)	0.65	0.2	(−2.9, 3.4)	0.14	0.88	(0.65-0.96)	0.5	5	1.5	14
GET% of VO_2_peak (GET%) [%]	75.1±11.1	(59.4-94.1)	69.9±13.2	(49.8-92.4)	0.09	5.2	(−16.3, 26.8)	0.15	0.57	(0.08-0.84)	7.1	10	19.7	27
O_2_ cost of work (ΔVO_2_/ΔP) [mL/min/W]	18.7±13.8	(5.7-51.1)	21.4±15.5	(4.9-60.4)	0.12	2.7	(−11.7, 17.1)	0.36	0.87	(0.66-0.96)	2.6	13	7.1	36
O_2_ pulse at VO_2_peak (O_2_pulse) [mL/beat]	10.2±4.6	(3.2-19.2)	10.3±3.8	(4.4-16.2)	0.73	0.1	(−3.3, 3.4)	0.17	0.92	(0.78-0.98)	0.5	5	1.3	13
V_E_ versus VCO_2_ slope (ΔV_E_/ΔVCO_2_) [L]	36.6±6.9	(20.4-47.6)	36.1±8.0	(18.4-52.2)	0.93	0.5	(−9.6, 10.6)	0.14	0.78	(0.44-0.92)	2.4	7	6.6	18
Deviation of work rate (RMSE_P_) [W]	8.8±10.3	(1.3-32.2)	8.5±7.7	(1.4-27.9)	0.93	0.3	(−21.9, 22.5)	1.28	0.28	(−0.32-0.70)	9.4	109	26.2	301

*Abbreviations*: *SD* Standard deviation, *MD* Mean difference, *LoA* Limits of agreement, *CoV* Coefficients of variation, *ICC* Intraclass correlation coefficient, *CI* Confidence interval, *SEM* Standard error of the measurement, *MDC* Minimal detectable change.

## Discussion

This is the first study to evaluate the test-retest reliability and repeatability of FC-RATE for assessment of exercise capacity early after severe stroke. The aims were: (1) to assess the clinical feasibility of FC-RATE for CPET in severely motor impaired individuals early after stroke, (2) to examine the ability of the concept to meet standard cardiopulmonary criteria for maximal exercise capacity, and (3) to assess the test-retest reliability and the repeatability of the approach.

### General observations

Despite rigorous exclusion criteria, only 90% of the sample completed both CLTs and 70% completed both IETs. Of the 6 subjects who dropped out during the study, only 2 were due to reasons based on uncontrollable factors such as cerebrospinal fluid leak and acute respiratory infection. The remaining 4 dropouts were caused by controllable factors such as abnormal gait pattern, tibia skin lesion, severe groin pain and lack of motivation. Skin lesions and severe groin pain due to inappropriate padding are preventable by extended familiarisation and padding procedures, whereas abnormal gait patterns due to spasticity and lack of motivation are difficult factors to control. Advanced control strategies might provide solutions for abnormal gait patterns and virtual reality approaches might facilitate motivation in the near future. Nevertheless, dropout rates were comparable with previous CPET studies in subacute stroke [3, 9–11].

The guidance of work rate for FC-RATE-based CPET was successful. RMSE_P_ values were below 10 W, which can be seen as acceptable based on previous pilot study results [39]. The approach presented here used work rate values of both legs together and does not consider the severe hemiplegia of the included subjects. As a result, subjects generally tend to exercise using the unaffected side more dominantly, which led to deviations from the predefined physiological gait pattern. The powered exoskeleton allowed the subjects to remain in an acceptable movement trajectory during FC-RATE. While the approach presented here aimed to recruit as much muscle mass as possible to provoke peak exercise capacity, the imbalance of muscular activation during FC-RATE might be relevant when applying the method in longitudinal training interventions, because continuous imbalance in the gait cycle might facilitate unwanted compensation patterns.

Overall, the findings present a promising method for CPET in severely motor impaired individuals after stroke, but important factors such as appropriate padding and force interactions between subject and robot must be well controlled to gain improvements towards clinical feasibility.

### Exercise capacity

For CLT, the difficulty of estimating the time constants for VO_2_ uptake kinetics (τ) in the transitions from rest to passive walking and passive walking to active walking was due to the inherent noisiness of the breath-by-breath data and the consequent poor signal-to-noise ratio. The approach presented here seems not appropriate to provide consistent CLT outcome values for the transition phases. Sudden onset of changes in BWS and walking pattern seem to have a strong impact on individual performance levels during conventional RATE that restricts valid assessment of VO_2_ uptake kinetics (τ) values. Cost of passive walking was comparable with previous studies using an identical setup [27, 31], whereas cost of active walking was considerably higher than previous results [39]. This difference might be caused by the inclusion of non-ambulatory subjects showing severe motor impairments (FAC 2.3 vs. FAC 1.1) in the present study.

With relative VO_2_peak values of 15.5 ± 4.9 mL/min/kg (51.6% of predicted VO_2_max), the results confirm that exercise capacity is seriously reduced within this group of severely motor impaired stroke survivors. Peak performance parameters during IET found in this study were slightly higher compared to previous trials using leg cycle ergometry, body weight supported treadmill training, and combined upper- and lower-limb ergometry [3, 4, 9–12]. This finding might be due to the introduction of treadmill exercise based CPET that has previously confirmed higher VO_2_peak values compared to leg cycle ergometry protocols [57]. Furthermore, the feedback-control approach presented in this study might have recruited additional muscle mass that provoked higher peak values compared to body weight supported treadmill training. Considering the inclusion of individuals with serious motor impairments and the comparable peak cardiovascular performance parameters, this study opens new perspectives regarding assessment of exercise capacity early after severe stroke.

The GET values observed in the current study (%GET =72.6 ± 12.2% of VO_2_peak) were in the upper range compared to sedentary healthy individuals (50-76% of VO_2_peak) [58], providing additional evidence of compromised exercise capacity in this population. ΔVO_2_/ΔP was higher compared to leg cycle ergometry and conventional treadmill exercise meaning that subjects required more oxygen for a given work rate level; this may be explained by a substantial amount of unaccounted work performed during the test [59–61]. Further research seems indicated to explore the impact on ΔVO_2_/ΔP while walking within different robotics-assisted systems.

Although the current study provides first evidence for clinical feasibility of using FC-RATE for CPET and promising results regarding assessment of maximal exercise capacity, the issue remains of whether the concept is able to meet traditional criteria for true maximal capacity. Only 2 subjects within this study showed a plateau in VO_2_ at the end of IET, traditionally considered the primary criterion for maximal aerobic effort. This finding is in line with previous studies [3, 10]. Even in healthy people, a plateau in VO_2_ response is not always seen during IET [62], therefore this criterion must be reconsidered for future analyses. With respect to the RER, only 1 subject achieved an RER value ≥1.15, and only 3 subjects (21%) reached RER ≥1.0. Compared to previous studies in subacute stroke which have shown mean RERpeak vales of 0.9 [4], 1.0 [3, 10], 1.02 [13], 1.1 [9], the results presented here are clearly in the lower range, but not unusual in this early phase after stroke. At least 5 subjects (36%) reached peak heart rate within 10 beats per minute of the age-predicted heart rate maximum, which is comparable with previous findings [3, 11]. Considering these results, most of the subjects appear not to have reached their maximal aerobic capacity. The main reason might be generalized and/or leg fatigue, because 93% of the subjects terminated the IET due to inability to maintain the target work rate, suggesting that impairments in strength, coordination, and sensorimotor control contribute to difficulties in producing high work rate levels. These findings are consistent with previous studies performing CPET in subacute stroke [4, 10]. Advanced control strategies of powered exoskeletons, i.e. adapting the movement trajectory to the subject’s needs (impairment level, hemiplegic side) and synchronising the treadmill inclination, might allow a more appropriate challenge progression to reach higher physical performance levels in this severely impaired population, and might provide closer approximations and comparisons to conventional treadmill based exercise testing procedures such as the Bruce or Balke protocols [59, 63].

Although most of the subjects appear to have performed in the submaximal range, the estimation of the work rate slope using MWC-W was shown to be successful. The approach implemented was able to reach peak performance within 8–12 minutes (tVO_2_peak =8.2 ± 2.6 min) during IET by defining the walking cadence at 60 steps/min while increasing the target work rate profile. This is an important finding for further research regarding the initial estimation of target work rate profiles for CPET in severely motor impaired populations.

Finally, considering the peak performance results and the low frequency of achieved criteria for VO_2_max of this study, in combination with previous study results on peak exercise capacity in subacute stroke, we hypothesise that the guidelines postulated for healthy populations may not be realistic for determination of true exercise capacity in the early stages after stroke [64–66].

### Test-retest reliability and repeatability

Most studies that examined test-retest reliability using CPET after stroke reported excellent relative reliability, whereas only Tang et al. revealed fair to good associations between trials [10, 14, 15, 17, 18]. The present study using a novel robotics-assisted treadmill-based method for assessment of exercise capacity revealed good to excellent relative reliability for the major peak cardiopulmonary performance parameters. There is only limited evidence so far on absolute reliability for CPET early after stroke. Compared to a previous study in chronic stroke using leg cycle ergometry that has shown SEM for relative VO_2_peak of 1 mL/min/kg (6%), our approach presented higher SEM values (2 mL/min/kg (13%)) [15]. Likewise, a previous study that used semi-recumbent leg cycle ergometry in subacute stroke with cognitive impairments yielded considerably lower MDC values (e.g. relative VO_2_peak: 0.97 mL/min/kg (4%) vs. 5.6 mL/min/kg (36%)) compared to our study [18].

While studies in healthy subjects and individuals with cardiac or respiratory disease have revealed high repeatability (CoV <0.10) [64, 67–69], the present study yielded considerably higher CoV for the major cardiopulmonary parameters (absolute VO_2_peak =0.28, relative VO_2_peak =0.25, Ppeak =0.28, HRpeak =0.10, absolute GET =0.14, relative GET =0.14, ΔVO_2_/ΔP =0.36, O_2_pulse =0.17, ΔV_E_/ΔVCO_2_ =0.14).

There was visual suspicion of heteroscedasticity for absolute VO_2_peak and Ppeak (Additional file 1); however, logarithmic transformation of the data (Additional file 2) did not change the relevant outcome variables, and thus appeared irrelevant fur further consideration. We hypothesise that the increase in bias along with higher work rate values is caused by large day-to-day variability in strength and/or coordinative capabilities. Although general factors that may contribute to variability in test-retest situations such as disease severity, patient instruction, time of day, testing procedure, and equipment, were well controlled in the protocol presented here, the novel approach might have led to additional confounding factors that could have influenced test-retest reliability and repeatability. A major factor was the high coordinative demand of the concept. Subjects not only had to walk (or pedal, as in earlier cycle ergometry studies); the challenge was to produce additional forces in the walking direction, where the exoskeleton restricted the movement. The results clearly indicated that the major reason for test termination was the inability to maintain P*mech, which led to the assumption that muscular and/or coordinative fatigue was the reason for test termination. Therefore, variation might be reinforced by day-to-day variability (normally ±3% [64]) and influenced by whether the test was maximal or not. This hypothesis is supported by the low RERpeak values reported in this study. More sophisticated strategies are required to reduce the load on the neuromuscular system while increasing cardiovascular stress during FC-RATE. This will possibly lead to a better approximation of true exercise capacity early after stroke and might improve the reliability and the repeatability of FC-RATE based CPET.

The approach presented here seems suitable for comparison of groups of stroke individuals or for assessment of group intervention effects in future studies, considering the range of between-group improvement in VO_2_peak of 12.6-34.8% [70–73] and Ppeak of 23.4-176.9% [72–74] after cardiovascular exercise in subacute stroke. Whether the absolute reliability and the repeatability reported are adequate to identify effectiveness of intervention programmes to improve exercise capacity should be part of future studies including larger sample sizes.

### Limitations

The major limitation of the current study is the small sample size, which may render the results underpowered. A sample size of at least 50 is generally seen as adequate for the assessment of the agreement parameter, based on a general guideline by Altman [75]. Considering the experimental approach of the method and the difficulty of implementing and performing CPET in the early stages after severe stroke, our sample of 20 subjects at onset was a realistic group size to evaluate first estimates from a clinical perspective.

The conventional sequencing of the test situations (CLT, CLT, IET, IET) might have led to practice effects. The severely impaired and early-post-stroke status of the individuals included in this experimental approach may justify this order to progressively increase the exercise intensity over time to control potential risks.

The present study protocol did not include ECG monitoring for reasons of practicability, which influenced the study sample by excluding individuals with cardiac risk factors for CPET. While around 75% of stroke survivors present some degree of cardiovascular disease [76], the sample of this study might not be representative. Nevertheless, there was an uncontrolled risk for cardiac events due to the absence of ECG despite the adherence to strict exclusion criteria for cardiovascular disease.

While the study protocol strictly controlled time of day for CPET, the tests were performed within 48 h or 72 h due to practical reasons. This time difference might have affected the recovery phase of the subjects, thus influencing the results.

## Conclusion

This study presents first evidence on reliability and repeatability for CPET in severely motor impaired individuals early after stroke using a feedback-controlled robotics-assisted treadmill. The results demonstrate good to excellent test-retest reliability and appropriate repeatability for the most important peak cardiopulmonary performance parameters. These findings have important implications for the design and implementation of cardiovascular exercise interventions in severely impaired populations. Future research needs to develop advanced control strategies to enable the true limit of functional exercise capacity to be reached and to further assess test-retest reliability and repeatability in larger samples.

## Electronic supplementary material

Additional file 1: **Bland-Altman plots.** The difference between trial 2 (T2) and trial 1 (T1) is plotted against the mean of T1 and T2 for the major outcome variables. (PDF 72 KB)

Additional file 2: **Bland-Altman plots (logarithmically transformed).** The difference between trial 2 (T2) and trial 1 (T1) is plotted against the mean of T1 and T2 for the major outcome variables. (PDF 71 KB)

## Abbreviations

ACSM: American College of Sports Medicine
BWS: Body weight support
CI: Confidence interval
CLT: Constant load testing
CoV: Coefficient of variation
CPET: Cardiopulmonary exercise testing
FAC: Functional ambulation classification
FC-RATE: Feedback-controlled robotics-assisted treadmill exercise
GET: Gas exchange threshold
HR: Heart rate
ICC: Intraclass correlation coefficient
IET: Incremental exercise testing
LoA: Limits of agreement
MD: Mean difference
MDC: Minimal detectable change
O_2_pulse: O_2_ pulse at VO_2_peak
Pmax: Maximal work rate
Pmech: Work rate
P*mech: Target work rate
RATE: Robotics-assisted treadmill exercise
RER: Respiratory exchange ratio
R_f_: Respiratory rate
RMSE_P_: Accuracy of work rate tracking (root-mean-square error)
RPE: Rating of perceived exertion
SDdiff: Standard deviation of the difference
SEM: Standard error of the measurement
VCO_2_: Carbon dioxide output
V_E_: Ventilation rate
VO_2_: Oxygen uptake
VO_2_peak: Peak oxygen uptake
ΔV_E_/ΔVCO_2_: VE versus VCO_2_ slope
ΔVO_2_/ΔP: Oxygen cost of work.
