# Supplementary figures and images for: Cardiopulmonary exercise testing early after stroke using feedback-controlled robotics-assisted treadmill exercise: test-retest reliability and repeatability
Source: J Neuroeng Rehabil. 2014 Oct 11;11:145. doi: 10.1186/1743-0003-11-145 (PMC4271449; doi:10.1186/1743-0003-11-145)

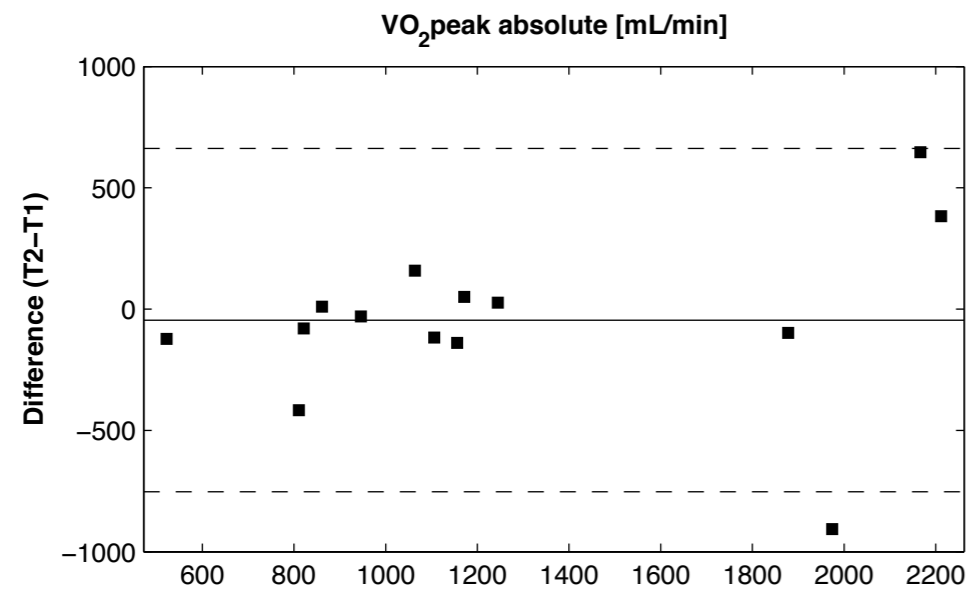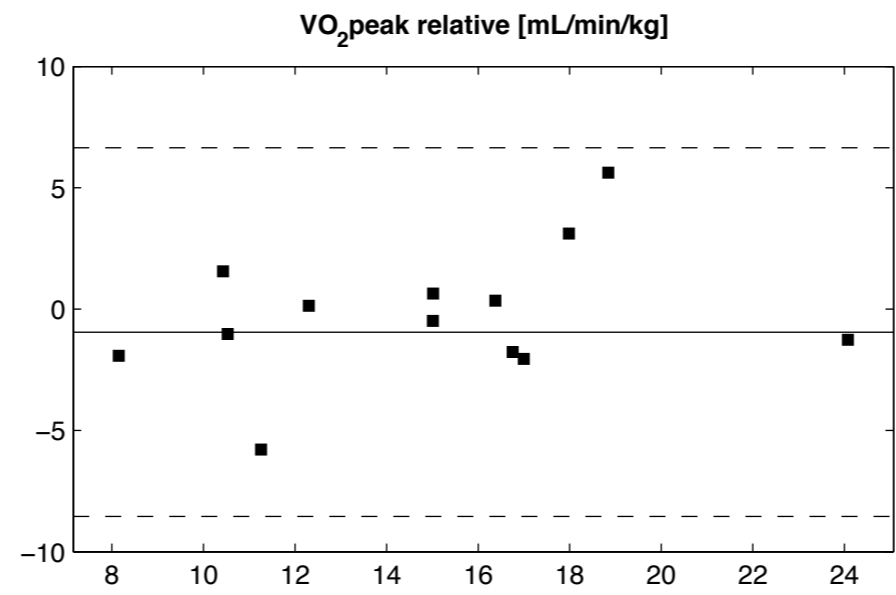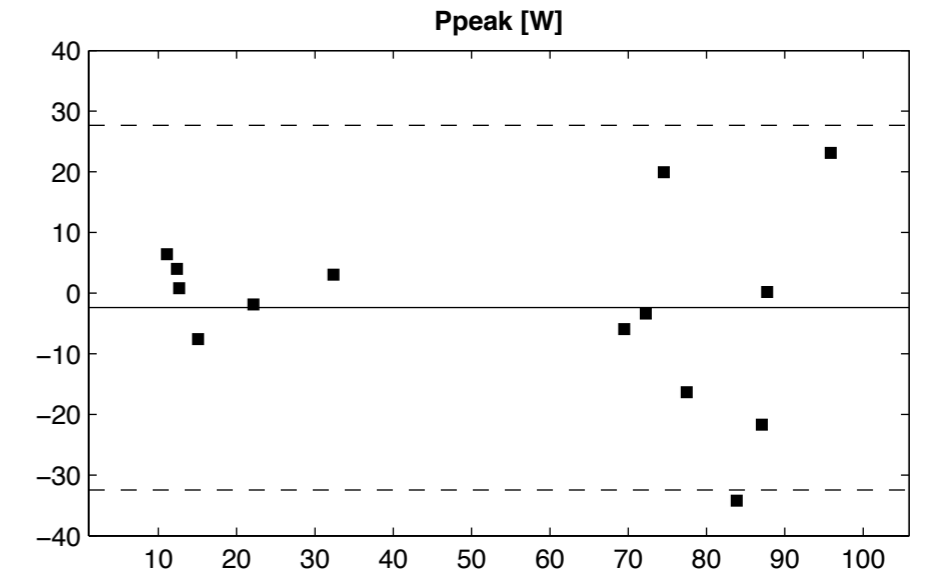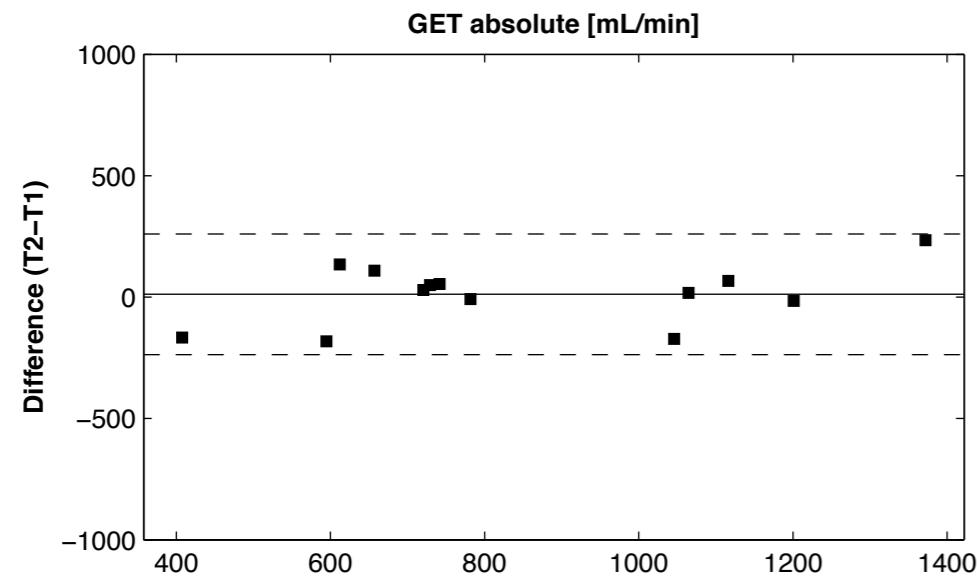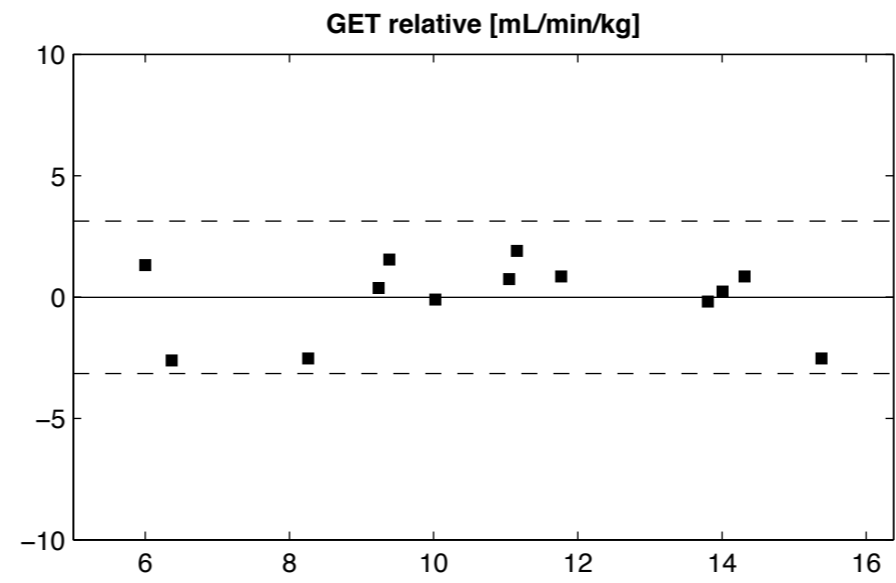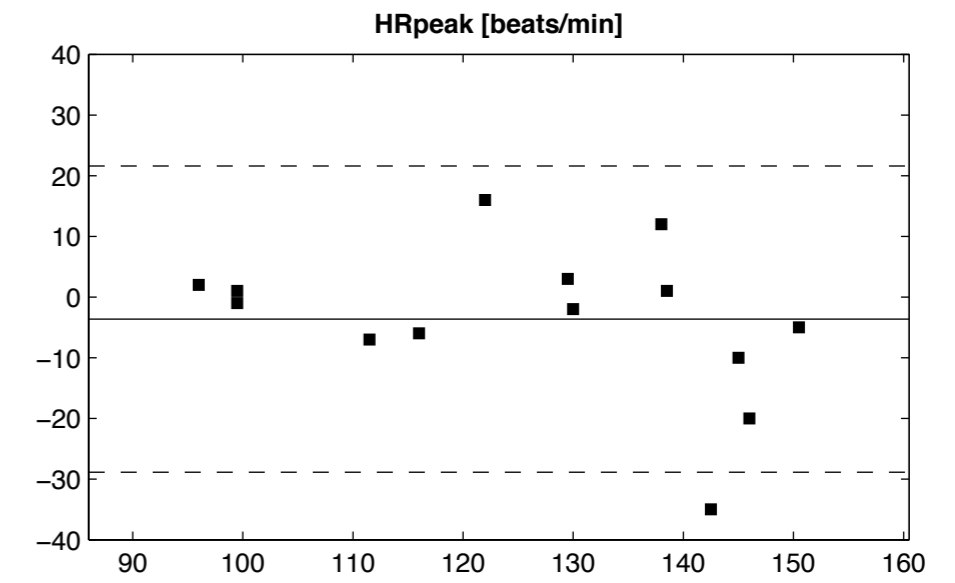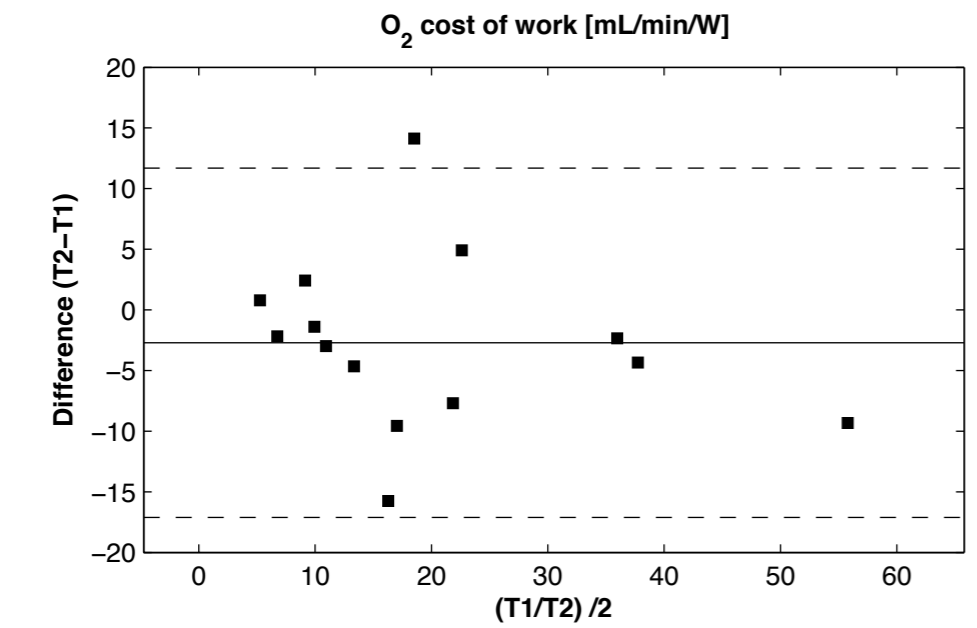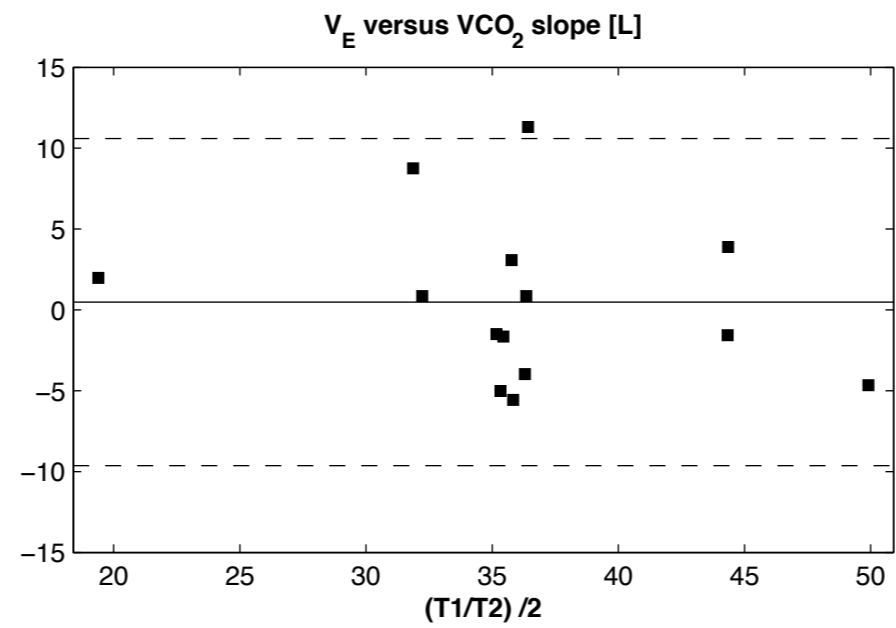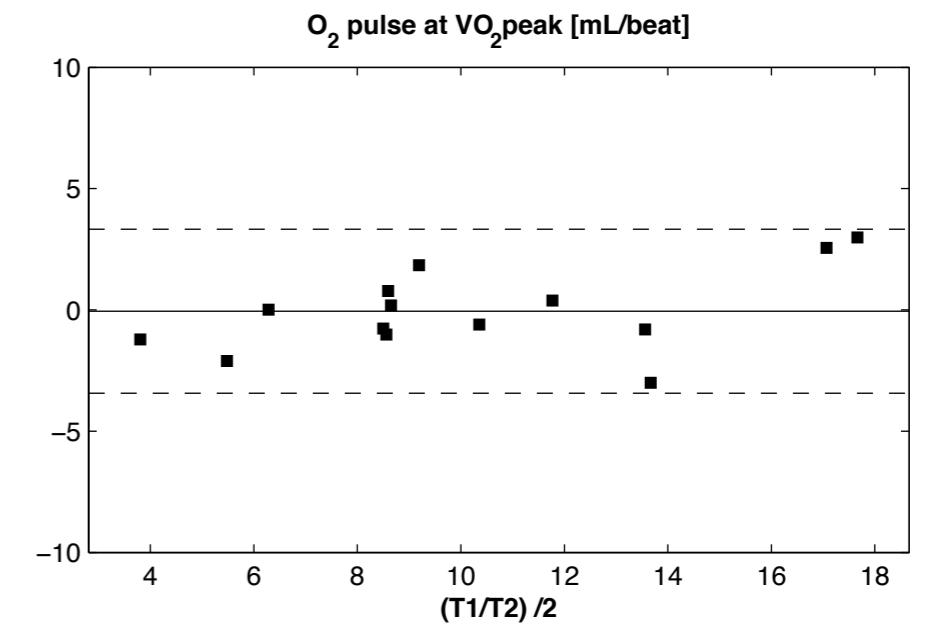

Supplement: Supplementary file 1 — Additional file 1: Bland-Altman plots. The difference between trial 2 (T2) and trial 1 (T1) is plotted against the mean of T1 and T2 for the major outcome variables. (PDF 72 KB) [file 12984_2014_677_MOESM1_ESM.pdf]

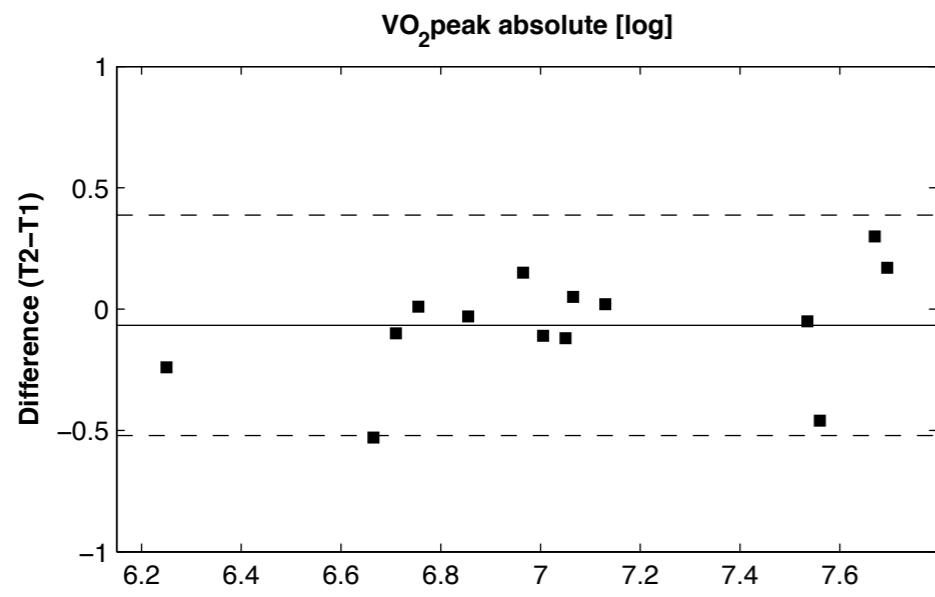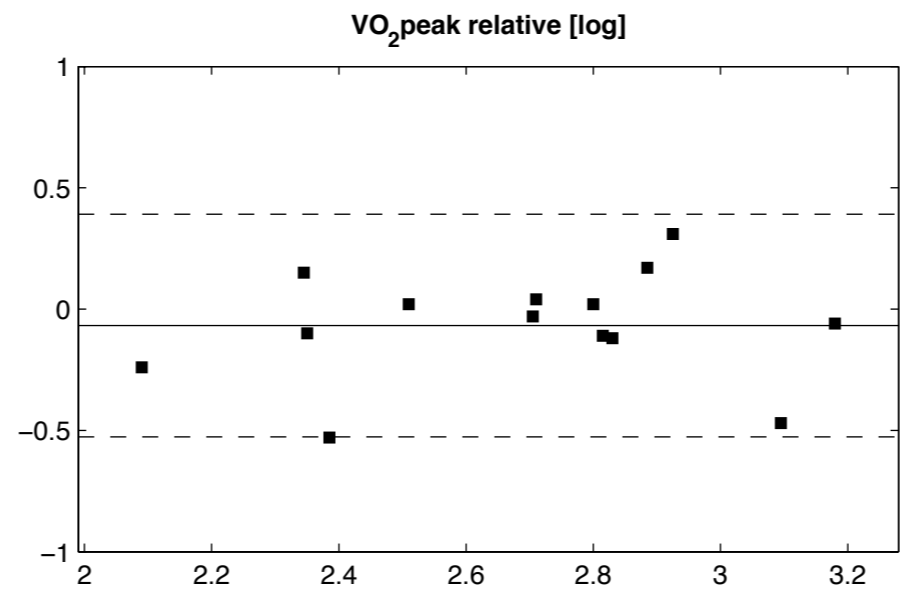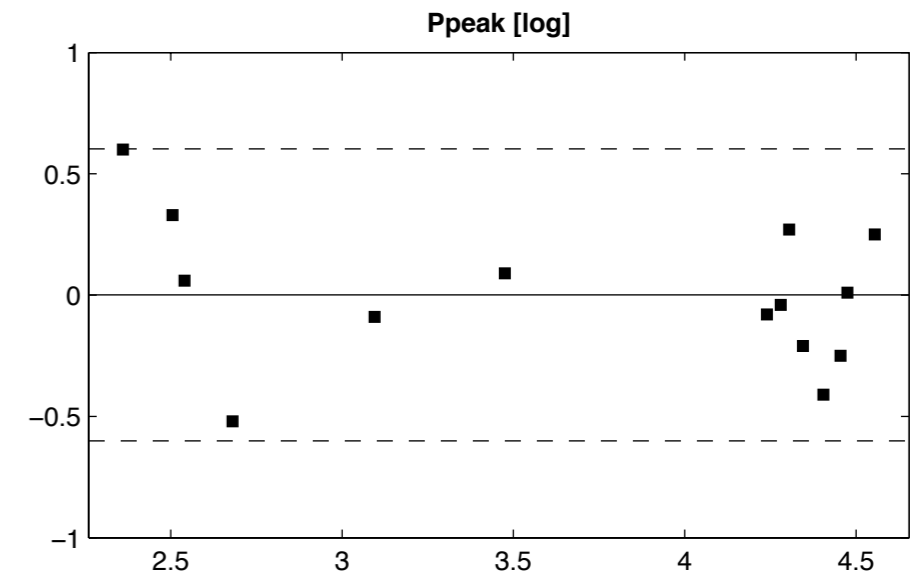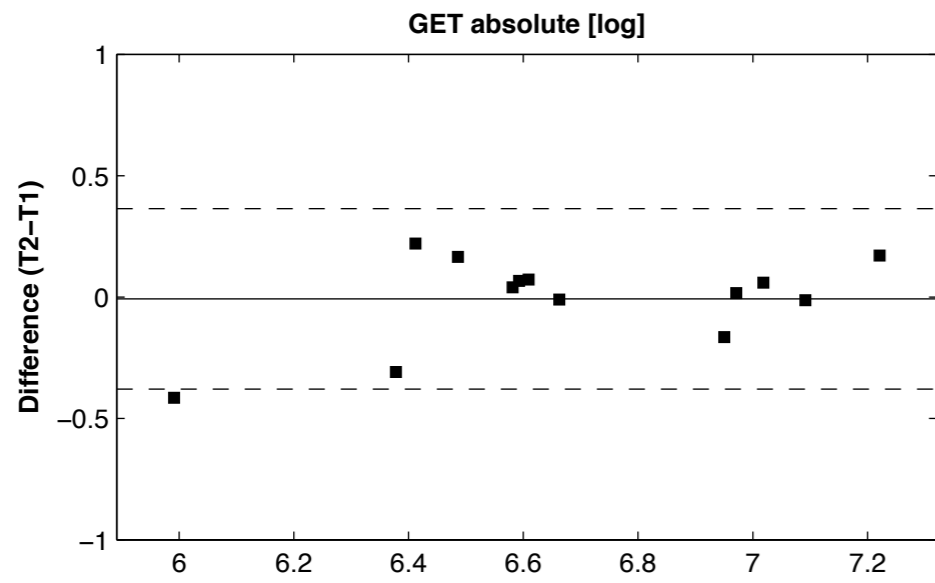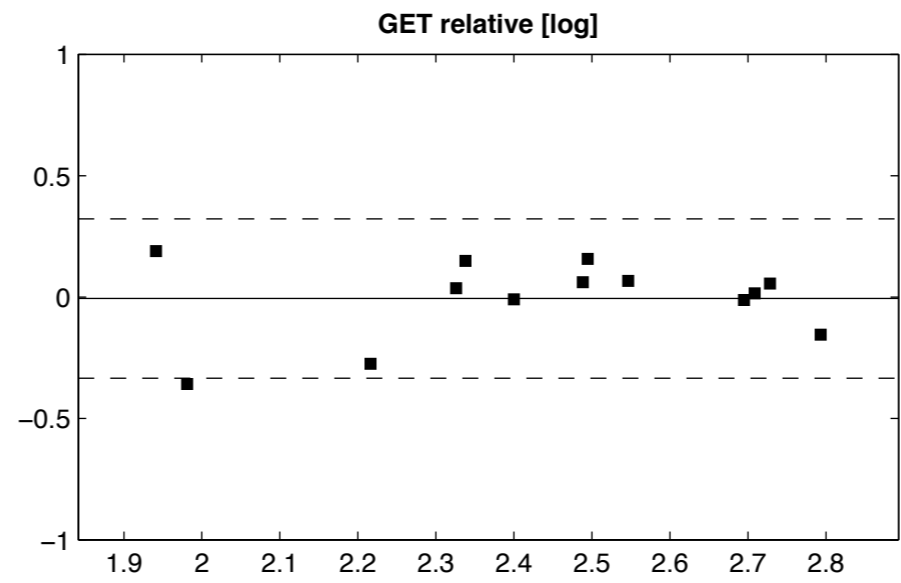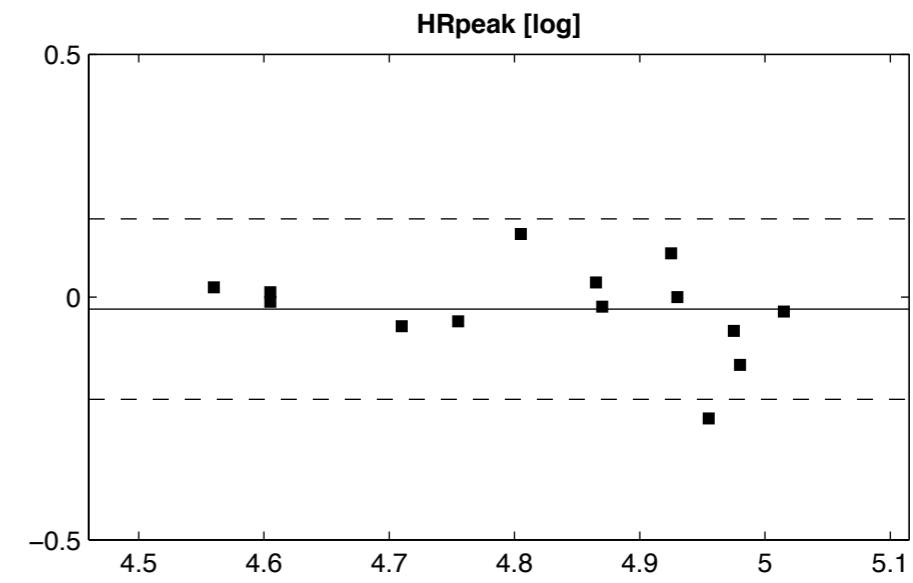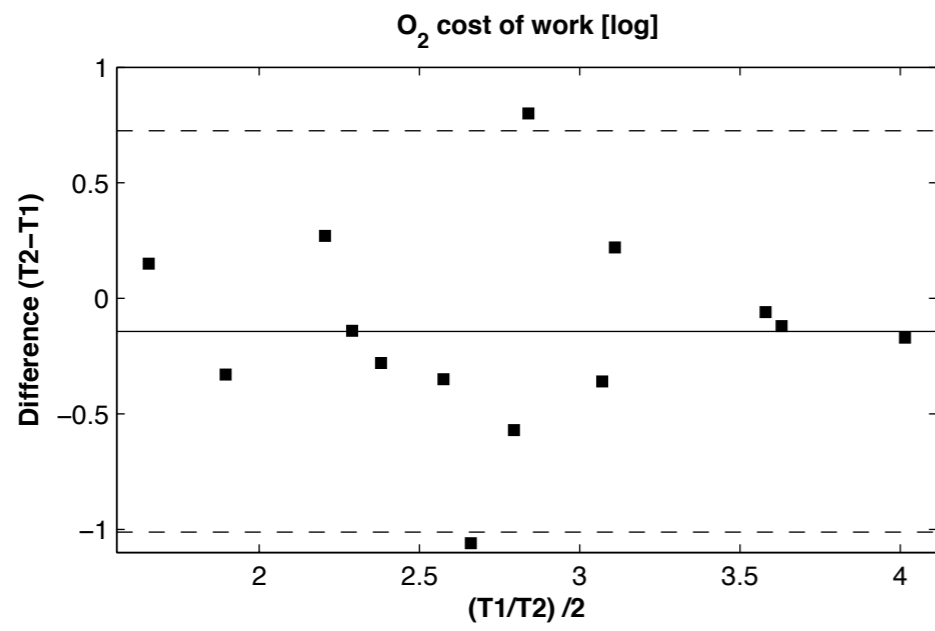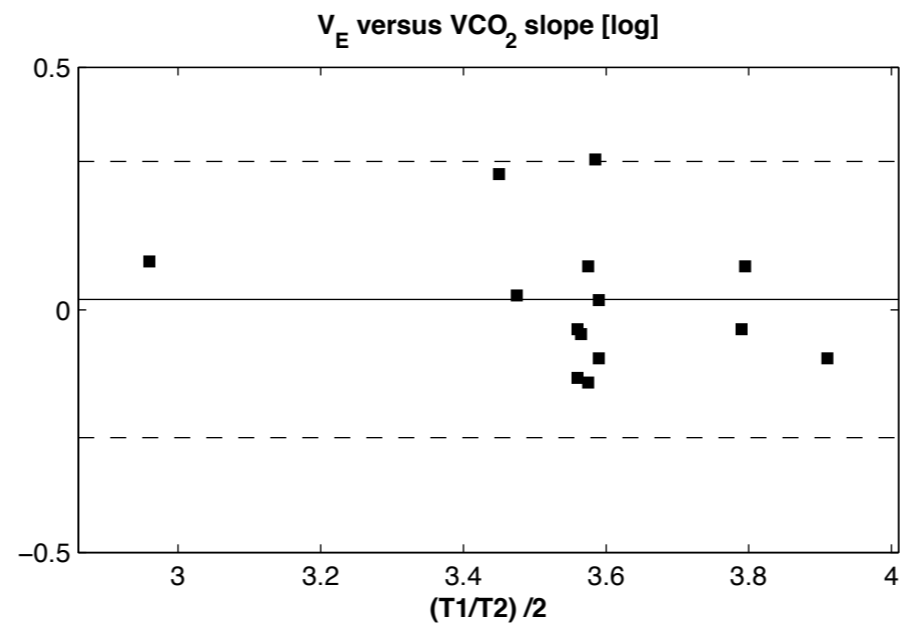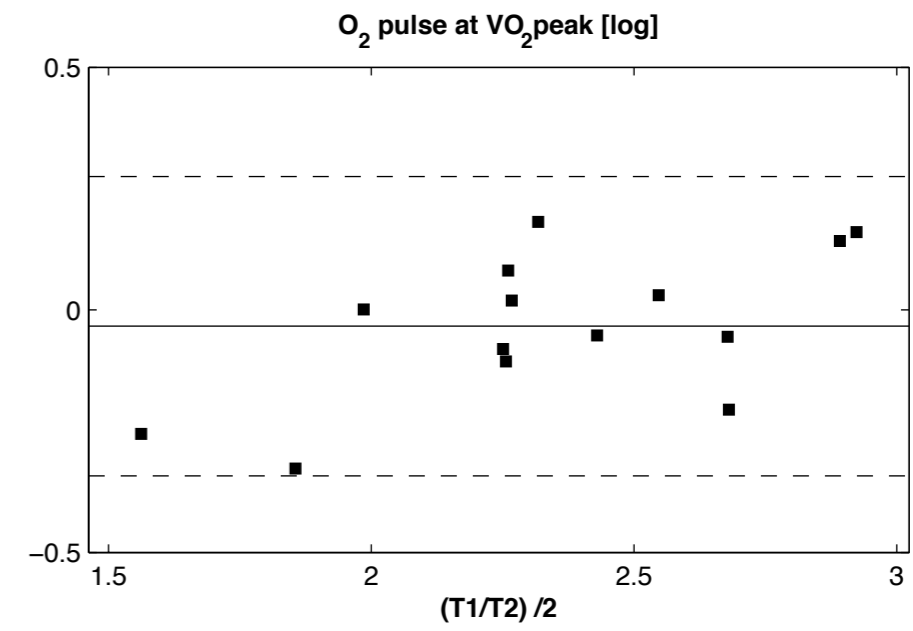

Supplement: Supplementary file 2 — Additional file 2: Bland-Altman plots (logarithmically transformed). The difference between trial 2 (T2) and trial 1 (T1) is plotted against the mean of T1 and T2 for the major outcome variables. (PDF 71 KB) [file 12984_2014_677_MOESM2_ESM.pdf]
